# Exploration of beta-arrestin isoform signaling pathways in delta opioid receptor agonist-induced convulsions

**DOI:** 10.3389/fphar.2022.914651

**Published:** 2022-08-11

**Authors:** Arryn T. Blaine, Yiming Miao, Jinling Yuan, Sophia Palant, Rebecca J. Liu, Zhong-Yin Zhang, Richard. M. van Rijn

**Affiliations:** ^1^ Department of Medicinal Chemistry and Molecular Pharmacology, Purdue University, West Lafayette, IN, United States; ^2^ Purdue Interdisciplinary Life Sciences Graduate Program, West Lafayette, IN, United States; ^3^ Purdue Institute for Drug Discovery, West Lafayette, IN, United States; ^4^ Purdue University Cancer Center, West Lafayette, IN, United States; ^5^ Purdue Institute for Integrative Neuroscience, West Lafayette, IN, United States

**Keywords:** biased signaling, seizure, ERK, mice, beta-arrestin 1, honokiol, PQR530, beta-arrestin 2

## Abstract

The δ-opioid receptor (δOR) has been considered as a therapeutic target in multiple neurological and neuropsychiatric disorders particularly as δOR agonists are deemed safer alternatives relative to the more abuse-liable µ-opioid receptor drugs. Clinical development of δOR agonists, however, has been challenging in part due to the seizure-inducing effects of certain δOR agonists. Especially agonists that resemble the δOR-selective agonist SNC80 have well-established convulsive activity. Close inspection suggests that many of those seizurogenic δOR agonists efficaciously recruit β-arrestin, yet surprisingly, SNC80 displays enhanced seizure activity in β-arrestin 1 knockout mice. This finding led us to hypothesize that perhaps β-arrestin 1 is protective against, whereas β-arrestin 2 is detrimental for δOR-agonist-induced seizures. To investigate our hypothesis, we characterized three different δOR agonists (SNC80, ADL5859, ARM390) in cellular assays and *in vivo* in wild-type and β-arrestin 1 and β-arrestin 2 knockout mice for seizure activity. We also investigated downstream kinases associated with β-arrestin-dependent signal transduction. We discovered that δOR agonist-induced seizure activity strongly and positively correlates with β-arrestin 2 efficacy for the agonist, but that indirect inhibition of ERK activation using the MEK inhibitor SL327 did not inhibit seizure potency and duration. Inhibition of the PI3K/AKT/mTOR signaling with honokiol but not PQR530, attenuated SNC80 seizure duration in β-arrestin 1 knockout, but honokiol did not reduce SNC80-induced seizures in wild-type mice. Ultimately, our results indicate that β-arrestin 2 is correlated with δOR agonist-induced seizure intensity, but that global β-arrestin 1 knockout mice are a poor model system to investigate their mechanism of action.

## 1 Introduction

**FIGURE 1:**
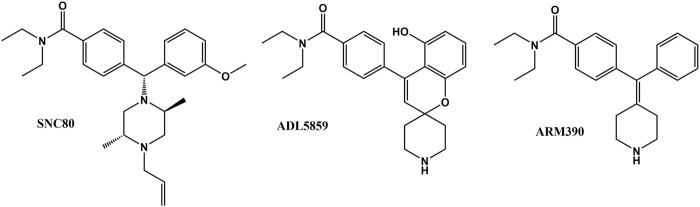
Chemical structure of SNC80, ADL 58589 and ARM 390.

The δ-opioid receptor (δOR) is a G-protein coupled receptor (GPCR) within the opioid family that has been considered as therapeutic target for the treatment of migraine, chronic pain, anxiety, and major depressive disorder (Jutkiewicz, 2006; Perrine et al., 2006; Pradhan et al., 2011; Pradhan et al., 2014; Gendron et al., 2016; Cahill et al., 2022). δOR agonists have also been proposed as promising treatments for alcohol use disorder, ischemia, and stroke (Schultz et al., 1997; Karck et al., 2001; Alongkronrusmee et al., 2018; Grant Liska et al., 2018; Sheng et al., 2018); particularly as δOR agonists generally display negligible abuse potential (Van Rijn et al., 2012; Hudzik et al., 2014; Conibear et al., 2020; Gutridge et al., 2021) and thus, would be safer alternatives relative to µ-opioid receptor drugs. Yet, in contrast to the µ-opioid and κ-opioid receptor, no δOR selective compounds have been approved for clinical use. In terms of drug development, one significant hurdle is that δOR agonists may induce seizures. This phenomenon has been described for multiple δOR ligands, both peptides (Haffmans and Dzoljic, 1983; Haffmans and Dzoljic, 1987; De Sarro et al., 1992) and small molecules. Especially the ‘SNC80-type’ of δOR agonists (BW373U86, SNC80, SNC162) have well-established convulsive activity (Comer et al., 1993; Broom et al., 2002a; Jutkiewicz et al., 2004; Jutkiewicz et al., 2005a; Jutkiewicz et al., 2005b). The ability of SNC80 to induce seizures is absent in δOR knockout mice, indicating an on-target effect (Broom et al., 2002b; Chung et al., 2015). Yet, multiple δOR selective agonists have been designed and characterized to specifically lack this seizurogenic effect (Saitoh et al., 2011; Saitoh et al., 2018; Conibear et al., 2020; Fossler et al., 2020; Gutridge et al., 2021). These findings hint at a concept of biased signaling, where a particular ligand activates a subset of signal-transduction pathways thereby promoting or avoiding specific cellular signaling and associated physiological consequences. Biased signaling, particularly as it relates to compounds preferring to signal via G-proteins over β-arrestin proteins has been reported for multiple GPCRs, including the opioid receptors (Gundry et al., 2017; Smith et al., 2018; Wootten et al., 2018). Close inspection suggests that many of the δOR agonists reported to have limited seizure activity do not recruit β-arrestin as effectively as the SNC80-type agonists that do induce seizures (Comer et al., 1993; Broom et al., 2002a; Jutkiewicz et al., 2004; Jutkiewicz et al., 2005a; Jutkiewicz et al., 2005b; Saitoh et al., 2011; Fenalti et al., 2014; Chiang et al., 2016; Conibear et al., 2020; Fossler et al., 2020; Gutridge et al., 2021). However, SNC80-induced seizures are retained in β-arrestin 2 knockout mice and enhanced in β-arrestin 1 knockout mice (Dripps et al., 2018; Vicente-Sanchez et al., 2018). This finding led us to hypothesize that perhaps β-arrestin 1 is protective against δOR seizures. To investigate our hypothesis, we characterized three different δOR agonists (SNC80, ADL5859, ARM390, Figure 1) in cellular assays and *in vivo* in wild-type and β-arrestin 1 and β-arrestin 2 knockout mice for seizure activity. ADL5859 is a δOR agonist that was deemed safe enough to enter phase II clinical trials, whereas ARM390 is considered a low-internalizing δOR agonist (Marie et al., 2003; Le Bourdonnec et al., 2008). We also investigated downstream kinases associated with β-arrestin-dependent signal transduction. Beyond the previously reported observation that β-arrestin 1 knockout mice show an increased susceptibility to δOR agonist-induced seizures, the mechanism by which these convulsions occur is poorly defined. We find that δOR agonist-induced seizure activity strongly and positively correlates with β-arrestin 2 efficacy for the agonist. More importantly, we have found that SNC80 increases pERK in the hippocampus of β-arrestin 1 knockout mice, but that indirect inhibition of ERK activation using the MEK inhibitor SL327 did not reduce seizure potency and duration. In contrast, Honokiol, a broad inhibitor of PI3K/AKT/mTOR signaling (Bai et al., 2003; Crane et al., 2009; Nagalingam et al., 2012; Pillai et al., 2015; Fan et al., 2018; Park et al., 2020), attenuated SNC80 seizure duration in β-arrestin 1 knockout, but not wild-type mice, nor did PQR530, a different modulator of PI3K/mTOR signaling block SNC80-induced seizures in β-arrestin 1 knockout mice. Ultimately, our study was able to eliminate a couple of signaling pathways as potential cause for δOR-induced seizures in wild-type mice, but we were unable to identify the underlying mechanism.

## 2 Materials and methods

### 2.1 Animals

Animal studies were carried out in accordance with the National Institutes of Health’s Guide for the Care and Use of Laboratory Animals. All animal protocols (#1305000864) describing the care and experimental use of animals were preapproved by the Purdue University Institutional Animal Care and Use Committee. Wild-type C57BL/6 male and female mice were purchased from Envigo (Indianapolis, IN) and β-arrestin 1 or 2 global knockout mice were bred in our facility (Purdue University, West Lafayette, Indiana, for source see (Robins et al., 2018). To reduce genetic drift, knockout mice have been rederived every 3 years by crossing the knockout mice with wild-type C57BL/6 mice from Envigo. Animals were kept in Purdue University’s animal facility, which is accredited by the Association for Assessment and Accreditation of Laboratory Animal Care, where mice were maintained at an ambient temperature of (21°C). Mice were group housed (2-5 mice) in plexiglass cages in ventilated racks in a room kept on a reversed 12L:12D cycle (lights off at 10.00, lights on at 22.00). Food and water were provided *ad libitum*. Adult male (19–25 g) and female (16–23 g) mice between 8–16 weeks old were used for our experiments. All purchased mice were acclimated to the animal facility for at least 1 week prior to experimentation.

### 2.2 Drug preparation

ADL5859 [30 mg/kg, Axon Medchem, Reston, VA] and ARM390 (10 mg/kg, Tocris, Thermo Fisher Scientific, Waltham, MA) were dissolved in distilled water, with ADL5859 administered intraperitoneally and ARM390 administered orally via gavage (OG); SNC80 (10 mg/kg, Tocris) was diluted in slightly acidic saline (pH 5–6) and administered intraperitoneally. Doses for each δOR agonist were equipotent for their ability to impact behavior through δOR (Pradhan et al., 2010; Nozaki et al., 2012). SL327 (50 mg/kg, Tocris) was diluted in a solution made of 5% dimethyl sulfoxide (VWR, Avantor, Radnor, PA); 10% Kolliphor EL (Millipore Sigma, Sigma Aldrich, St. Louis, MO); and 85% of 0.9% saline and administered subcutaneously (SC) (Ko et al., 2021). Honokiol (40 mg/kg, MedChemExpress, Monmouth Junction, NJ) and PQR530 (25 mg/kg, MedChemExpress) were diluted in a solution made of 5% dimethyl sulfoxide (VWR, Avantor, Radnor, PA); 10% Kolliphor EL; and 85% of 0.9% saline; and Honokiol was also made at 80 mg/kg in a solution of 10% dimethyl sulfoxide; 10% Kolliphor EL; and 80% of 0.9% saline. PQR530 and honokiol were administered orally via gavage (Godugu et al., 2017). Scopolamine methyl bromide (1 mg/kg, Millipore Sigma) was dissolved in water and administered intraperitoneally (IP) (Navarro Mora et al., 2009) to serve as muscle relaxant. Despite the scopolamine use, we still had two mice that may have bit their tongue during a seizure, and which were euthanized at the first sign of this, before their trial ended.

### 2.3 Seizure testing and scoring

All testing was done between 10.00 and 18.00 during the mouse’s active cycle. Mice were moved to a room with regular light and allowed to acclimate to the testing room for at least 15 min prior to testing. Mice were weighed and habituated to injections the day before seizure testing. Prior to test drug administration, animals were pretreated with scopolamine methyl bromide (1 mg/kg, IP) (Turski et al., 1984). For studies analyzing SNC80, ADL5859 and ARM390, within each genotype (wild-type, β-arrestin 1 knockout and β-arrestin 2 knockout) an *n* = 6 was used comprising of 3 male and 3 female mice being tested for each drug (total *n* = 54). One exception is that 5 male and 5 female β-arrestin 1 knockout were tested with SNC80; this was because these tests were used as a positive control. For experiments testing SL327 and SNC80, 3 male and 3 female wild-type and β-arrestin 1 knockout mice were tested with SL327 + SNC80, and only SL327 (total *n* = 24). For experiments testing 40 mg/kg Honokiol and SNC80, 3 male and 3 female β-arrestin 1 knockout mice were tested with Honokiol + SNC80, and only Honokiol (total *n* = 12). For experiments testing 80 mg/kg Honokiol and SNC80, 3 female and 2 male β-arrestin 1 knockout mice were tested (total *n* = 5). 3 male and 3 female β-arrestin 1 KO mice were tested with PQR530 and SNC80 (total *n* = 6). 6 female wild-type mice were tested with Honokiol + SNC80 and 6 female wild-type mice were tested with only SNC80 (total *n* = 12). After last injection, mice were immediately placed in a clear, plexiglass cage and recorded for 60 or 180 min. Videos footage was analyzed using the Modified Racine score (Luttjohann et al., 2009), where every 3 min of video was scored. Score of 1: sudden behavioral arrest and/or motionless staring; Score of 2: facial jerking with muzzle or muzzle and eye; Score of 3: neck jerks; Score of 4: clonic seizures in a sitting position; Score of 5: convulsions including clonic and/or tonic–clonic seizures while lying on the belly and/or pure tonic seizures; Score of 6: convulsions including clonic and/or tonic–clonic seizures while lying on the side and/or wild jumping. We did not observe any scorable seizure activity for vehicle (water, acidified saline, data not shown). Seizures in one male and one female mouse co-treated with SNC80 and SL327 developed were severe enough that they displayed blood in/near their mouth. Both mice were immediately euthanized and for the remainder of the trial these mice were assigned a seizure score that was equal to the final recorded score prior to euthanasia.

### 2.4 Preparation of tissue homogenates

Separate cohorts of β-arrestin 1 knockout and wild-type mice were utilized for western blot studies. Male and female β-arrestin 1 KO mice were administered 10 mg/kg SNC80 (*n* = 3 per sex) or saline (*n* = 3 per sex, total *n* = 6). Tissue was collected as previously described (Ko et al., 2021) 30 min post SNC80 administration. Based on our previous studies, we have particularly chosen carbon dioxide asphyxiation over other euthanasia methods that may potentially increase basal ERK1/2 activity in the brain (Ko et al., 2019). The entire hippocampus (dorsal and ventral) was dissected as previously described (Spijker, 2011), and flash frozen in dry ice. This procedure was chosen to avoid reproducing the data generated by Ko et al., for which the dorsal and ventral hippocampus were separately collected via micropunch (Ko et al., 2021). Collected tissue was further homogenized in cold RIPA buffer mixed with HALT™ Protease and Phosphatase Inhibitor Cocktail (Thermo Fisher Scientific) via titrating brain tissue in a 1 ml syringe (Luer slip tip, graduated, Sigma-Aldrich, St. Louis, MO) with 25-gauge needle (BD General Use and Precision Glide Hypodermic needle, Thermo Fisher Scientific, Waltman, MA). Samples were further prepared based on established protocols (Ko et al., 2019; Ko et al., 2021).

### 2.5 SDS-page and western blot

Samples (20 µL containing 10 µg protein) were loaded per well of a NuPage 4–12% Bis-Tris gradient gels (#NP0336BOX, Thermo Fisher Scientific), and the SDS-Page gel was subsequently transferred to nitrocellulose membranes (#1620115, BioRad) and Western blot was carried out using the following primary rabbit anti-Phospho-p44/42 MAPK (Erk1/2) (Thr202/Tyr204) (D13.14.4E) XP^®^ (Cell signaling, MA, catalog 8544S, 1:1,000), mouse anti-p44/42 MAPK (Erk1/2) (L34F12) (Cell Signaling, catalog 4696S, 1:1,000), mouse anti-GAPDH (1D4) (Novus Biologicals, CA, catalog 221, 1:1,000) and Li-Cor secondary antibodies: anti-mouse IRdye 680LT (Li-Cor, Lincoln, NE, catalog 926–68020, 1:5,000) and anti-rabbit IRDye 800CW (catalog 926–32211, 1:5,000). Prepared samples were scanned using the Li-Cor Odyssey^®^ CLx Scanner (Li-Cor). For statistical analysis, all ERK bands were normalized to GAPDH.

### 2.6 Cell culture and cellular assays

U2OS-δOR-β-arrestin 1 cells (DiscoverX, Fremont, CA RRID:CVCL_LA96) and Chinese hamster ovarian CHO-δOR-β-arrestin 2 cells (DiscoverX, RRID:CVCL_KY68) were cultured as recommended by the manufacturer and maintained at 37 °C/5% CO_2._ For β-arrestin recruitment assays, U2OS-δOR-β-arrestin 1 cells or CHO-δOR-β-arrestin 2 cells were plated in low -volume 384 well plates at a concentration of 2,500 cells per well (10 µL) a day prior to running the assay. The next day, cells were stimulated with 2.5 µL of drug solution for 90 min at 37 °C/5% CO_2_ as previously described (Cassell et al., 2019; Ko et al., 2021). Following the manufacturer’s protocol, cells were then incubated with 6 µL of PathHunter assay buffer (DiscoverX) for 60 min at room temperature. The FlexStation3 plate reader (Molecular Devices, Sunnyvale, CA) was used to measure luminescence for all assays. Leu^5^-enkephalin (Sigma Aldrich) was used as reference molecule.

### 2.7 Data and statistical analysis

All data is presented as means ± standard error of the means (SEM). Data analysis was performed using GraphPad Prism 9 software (GraphPad Software, La Jolla, CA). Seizure assays were analyzed for statistical significance using student t-test, one-way or two-way Analysis of Variance (ANOVA) depending on the number of treatment groups and independent variables. If a significant deviation of the mean *p* < 0.05 was identified, and an appropriate post-hoc analysis was performed as recommended by Prism (generally Tukey’s). For *in-vitro* assays, nonlinear regression was used to determine pEC_50_ (β-arrestin recruitment assays). Each data point for β-arrestin recruitment assays was performed as a technical duplicate in at least three biological triplicates. For each experiment, a positive control or reference compound was used to normalize the data and determine the log bias value.

## 3 Results

### 3.1 SNC80, ADL5859 and ARM390 produce weak, but not non-existent seizure activity in wild-type mice

**FIGURE 2:**
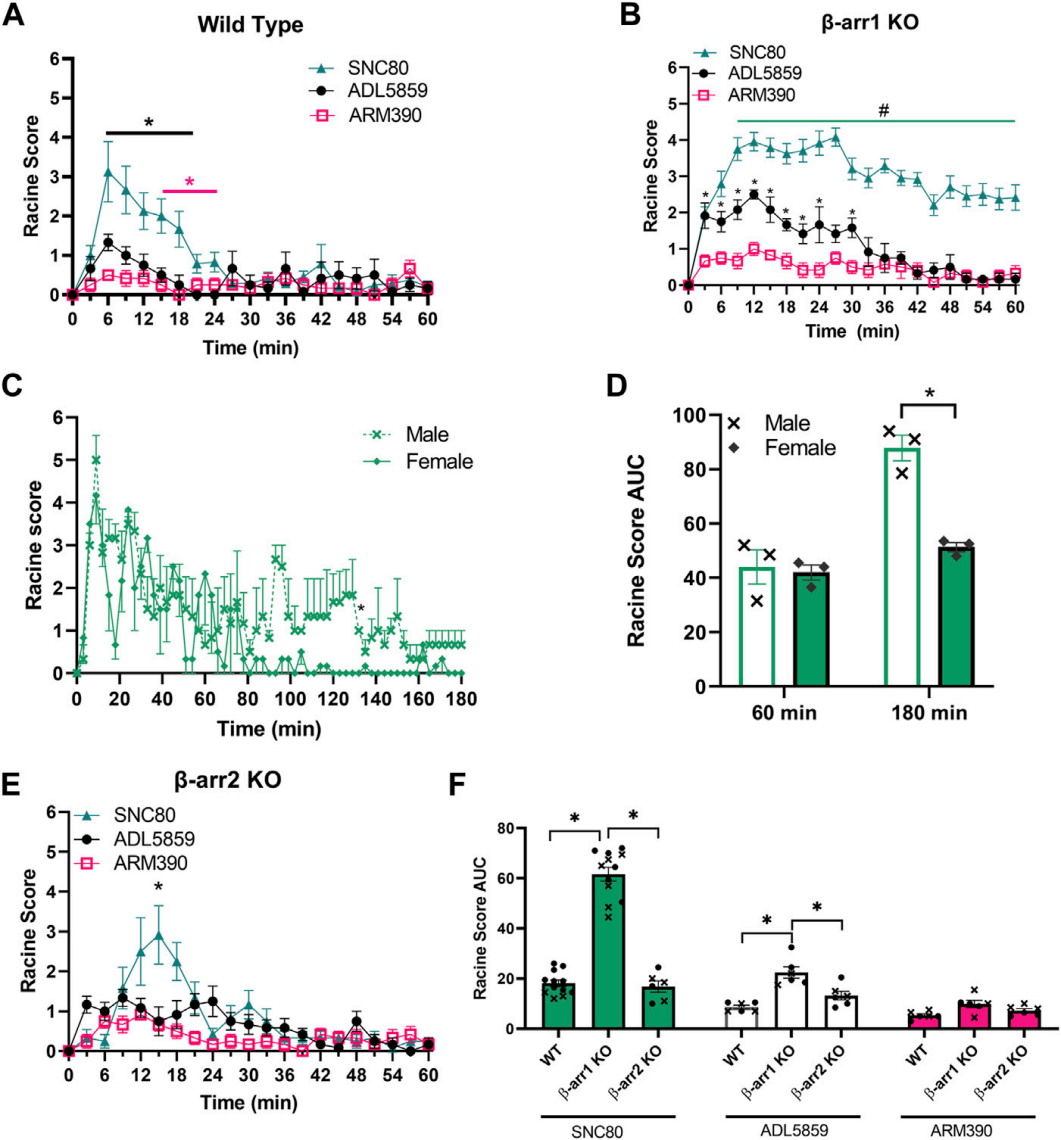
β-arrestin 1 knockout mice display enhanced seizure activity for δOR agonists relative to wild-type and β-arrestin 2 knockout mice. **(A) **Seizure activity over 1 h period for equi-antinociceptive doses of SNC80 (10 mg/kg i.p.), ADL 58589 (30 mg/kg, i.p.) and ARM 390 (10 mg/kg p.o.) in wild-type mice. **(B)** Seizure activity in β-arrestin 1 KO mice. **(C)** Seizure activity for SNC80 in male and female β-arrestin 1 KO mice over a 3-h period. **(D)** Area under the curve (AUC) for seizure activity induced by SNC80 (10 mg/kg i.p.) in male and female mice in β-arrestin 1 KO mice over 60 and 180 min. **(E)** seizure activity in β-arrestin 2 KO mice **(F)** Area under the curve for SNC80, ADL 58589 and ARM 390 in wild-type, β-arrestin1 KO, and β-arrestin 2 KO mice. Racine score data is shown 60 minutes post-injection. Equal number of males and females were tested for each drug (n = 6 total, except n = 10 for SNC80 in β-arrestin 1 KO mice). *p < 0.05. In **(G)** male mice are indicated with a cross whereas female mice are depicted as circles.

While it has been well-established that the δOR agonist SNC80 induces seizures (Jutkiewicz et al., 2005b; Chung et al., 2015; Vicente-Sanchez et al., 2018), other δOR agonists, such as the clinical trial candidate ADL5859 and ARM390 reportedly have no or limited seizure activity in wild-type mice (Chung et al., 2015). Importantly though, not all seizure activity results in a tonic-clonic seizure. Seizures were scored using the 6-point modified Racine scale, in which the lowest score describes facial clonus and behavioral arrest, paired with unique electroencephalogram (EEG) presentation (Luttjohann et al., 2009; Van Erum et al., 2019). Thus, we first assessed seizure activity for equi-antinoceptive doses (Pradhan et al., 2010; Nozaki et al., 2012) of SNC80 (10 mg/kg), ADL5859 (30 mg/kg) and ARM390 (10 mg/kg) in wild-type mice (Figure 2A). 2-Way ANOVA analysis revealed significant differences in Racine scores in wild-type mice between the three agonists [repeated measure 2-way ANOVA, agonist effect, F (2,21) = 30.41, *p* < 0.0001 and a time × agonist interaction effect F (40,420) = 2.39, *p* < 0.0001]. We noticed that SNC80 is the only agonist of the three to reach Racine scores higher than 2 throughout the 60 min.

### 3.2 Seizure activity for SNC80 and ADL5859 is enhanced in β-arrestin 1 knockout mice

SNC80 has also previously been shown to induce enhanced seizure activity in β-arrestin 1 knockout male and female mice (Vicente-Sanchez et al., 2018). Given that ADL5859 and ARM390 are based on the SNC80 scaffold, and that we could discern weak (Racine score ∼1) seizure activity for these compounds in wild-type mice, we hypothesized that ARM390 and ADL5859 are not completely devoid of seizure-inducing potential and would induce some degree of seizure activity in β-arrestin 1 knockout mice. Our seizure studies revealed that SNC80, ADL5859 and ARM390 all produced seizure activity in β-arrestin 1 knockout mice (Figure 2B). When comparing impact of the three drugs, we see that SNC80 induces significantly higher Racine scores, reaching as high as scores of 6 in individual mice, compared to both ADL5859 and ARM390, with neither causing average scores above 3 [repeated measure 2-way ANOVA, agonist effect, F (2,21) = 111.4, *p* < 0.0001 and a time × agonist interaction effect F (40,420) = 3.73, *p* < 0.0001]. Tukey’s multiple comparison found that the SNC80-induced seizure effect was significantly different from ARM390 at each time point, and different from ADL5859 starting at 9 min; ADL5859-induced seizure effect differed from ARM390 for the first 30 min post administration.

### 3.3 Both potency and duration of SNC80 induced seizures is enhanced in β-arrestin 1 KO mice and introduces sex differences

Seizure activity for our panel of δOR agonists in wild-type mice returned to zero within 1 h, and thus we stopped scoring wild-type mice after 60 min. However, in the β-arrestin 1 knockout mice, mice exposed to SNC80 still exhibited pronounced seizure activity beyond the 1-h mark. Therefore, we decided to extend our recording and analysis of SNC80-induced seizure activity in β-arrestin 1 knockout mice from 1 to 3 h instead (Figure 2C). We found that it took female mice nearly 1.5 h to return to baseline, whereas male mice showed apparent seizure activity well beyond the 2-h mark, up until the 3-h time point. Notably, while there was no apparent sex-difference for the first hour, beyond this timepoint, male and female mice display a significant sex difference (repeated measure 2-way ANOVA, overall sex effect *p* = 0.002, F (1, 8) = 20.24, with Sidak’s multiple comparison indicating no difference between male and female over the first 60-min period post administration but a significant effect when measuring seizure activity across the 180-min period post administration (Figure 2D).

### 3.4 δOR agonist-induced seizure activity in β-arrestin 2 KO mice is not significantly different from wild-type mice

Based on the confirmed finding that δOR agonists exhibit enhanced seizure activity in β-arrestin 1 knockout mice, we hypothesized that β-arrestin 1 may be protective against seizures. Under this hypothesis, we predicted that β-arrestin 2 knockout mice, that lack β-arrestin 1, would have milder seizures than wild-type mice. When we analyzed β-arrestin 2 knockout mice seizure responses to SNC80, ADL5859 and ARM390, seizure activities are similar to wild-type mice where SNC80 induced highest level of Racine scores but statistically significantly different from ADL5859 and ARM390 at the 15-min timepoint (RM 2-way ANOVA, F (2,15) = 8.11, *p* < 0.01 and an overall time × agonist interaction effect *p* < 0.0001, F (40, 300) = 3.25 (Figure 2E). To compare results from all genotypes of mice in response to the three δOR agonists tested, we measured area under the curve for each agonist seizure activity in each genotype (Figure 2F). We found a significant genotype effect *p* < 0.0001, F (2, 49) = 93.87. β-arrestin 1 knockout mice displayed significantly higher Racine scores compared to wild-type and β-arrestin 2 knockout mice in response to SNC80 and ADL5859; while there is no significant difference among the genotypes for ARM390, β-arrestin 1 knockout mice continue to show slightly higher scores compared to wild-type and β-arrestin 2 knockout mice. There is no significant difference between wild-type and β-arrestin 2 knockout mice in seizure response to any of the drugs tested. Altogether, these results demonstrate that β-arrestin 1 knockout mice specifically have higher propensity towards convulsive behavior in response to δOR agonists, compared to wild-type or β-arrestin 2 KO mice.

### 3.5 δOR agonist-induced seizure activity is strongly and positively correlated with β-arrestin 2 recruitment efficacy

**FIGURE 3:**
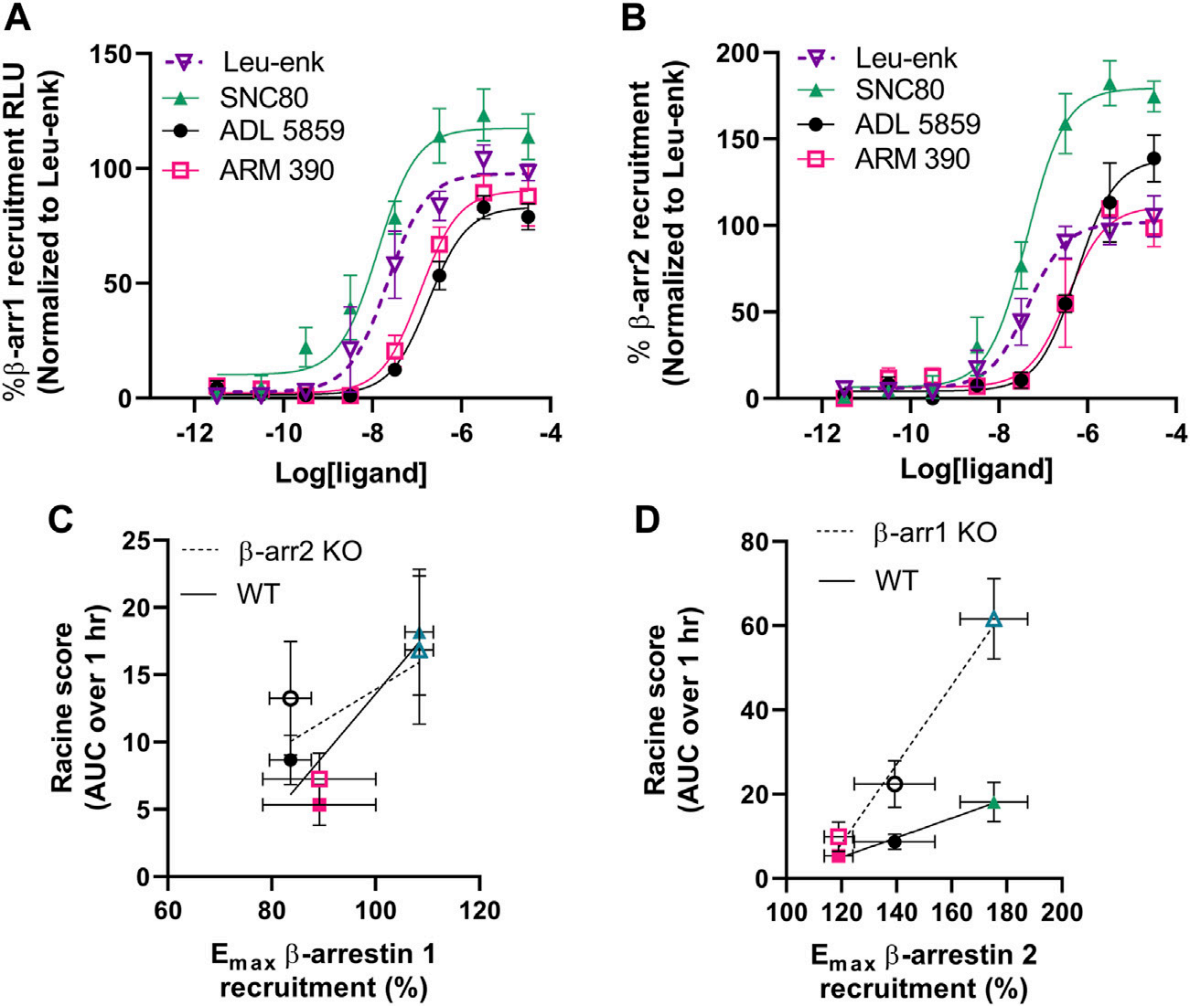
δOR agonist-induced seizure efficacy strongly and positively correlates with β-arrestin 2 recruitment efficacy. Concentration-dependent recruitment of β-arrestin 1 **(A)** or β-arrestin 2 **(B)** recruitment to δOR induced by SNC80, ADL5859 and ARM390. Data is normalized to the endogenous agonist peptide Leu^5^-enkephalin (Leu-Enk). Correlation plot for Racine score (60 min AUC) for 10 mg/kg SNC80 (n=12 for wild-type and β-arrestin 1 knockout mice, and n = 6 for β-arrestin 2 knockout mice), 30 mg/kg ADL 5859 (n = 6) and 10 mg/kg ARM 390 (n = 6) obtained in wild-type and β-arrestin 2 knockout mice **(C)** and in wild-type and β-arrestin 1 knockout mice **(D)** mice in correlation with its β-arrestin 1 **(C)** or β-arrestin 2 **(D)** recruitment E_max_. *p < 0.05. Symbols and colors in **(C,D)** correspond with the agonists as described in **(A,B)**. E_max_ values and the standard deviation represented are as depicted in Table 1.

From our initial characterization of δOR agonist-induced seizure activity we noted a stratification in the seizure intensity between ARM390, ADL5859 and SNC80. We hypothesized that the ability of these δOR agonists to induce seizures may be attributed to their ability to recruit β-arrestin. Therefore, we characterized the pharmacology of SNC80, ADL5859 and ARM390 for their potency and efficacy in recruiting β-arrestin 1 and β-arrestin 2 relative to the endogenous agonist Leu-enkephalin. While all three compounds recruit both isoforms of β-arrestin, SNC80 was the most efficacious and potent drug in recruiting both β-arrestin 1 and β-arrestin 2 (Figures 3A,B; Table 1). ADL5859 and ARM390 displayed similar potencies for recruiting both β-arrestin proteins; but slightly different efficacies with ARM390 being more efficacious for β-arrestin 1 (Figure 3A) and ADL5859 being more efficacious for β-arrestin 2 (Figure 3B), in agreement with published data (Chiang et al., 2016). We had previously identified that modulation of alcohol intake in mice by δOR agonists is strongly correlated with β-arrestin recruitment E_max_ (Chiang et al., 2016), so we next determined if there is a relationship between our tested δOR agonists’ β-arrestin recruitment efficacy (E_max_) and the intensity of seizures they induced at the equi-analgesic doses of 10 mg/kg for SNC80 and ARM390 and 30 mg/kg ADL5859. We found that there exists a weak to moderate correlation between β-arrestin 1 recruitment E_max_ and Racine score achieved by the mice over the 60 min (AUC) post dosing (Pearson *r* ^2^ squared for β-arrestin 2 KO: 0.40; and for wild-type: 0.80) (Figure 3C). Alternatively, there is a strong positive correlation between β-arrestin 2 recruitment efficacy of the drug, and the Racine score achieved by the mice over the 60 min (AUC) after agonist exposure (Pearson *r* ^2^ squared for β-arrestin 1 knockout: 0.98; and for wild-type: 0.99) (Figure 3D).

**TABLE 1:** Average potency (pEC_50_) and Emax (α) values with standard error of the mean (SEM) for δOR agonists in recruiting β-arrestin 1 and β-arrestin 2. All agonists were tested *n* ≥ 3.

	δOR–β-arrestin 1			δOR–β-arrestin 2		
Agonist	pEC_50_	EC_50_ (nM)	α (%)	pEC_50_	EC_50_ (nM)	α (%)
SNC80	7.77 ± 0.1	12	108 ± 3	7.51 ± 0.2	43	175 ± 12
ADL5859	6.70 ± 0.1	182	84 ± 4	6.27 ± 0.1	501	139 ± 15
ARM390	6.99 ± 0.2	116	89 ± 11	6.40 ± 0.2	346	119 ± 5
Leu-Enk	7.79 ± 0.6	22	100	7.70 ± 0.3	37	100

### 3.6 Inhibition of SNC80-induced ERK activation does not reduce seizure response in β-arrestin 1 knockout or wild-type mice

**FIGURE 4:**
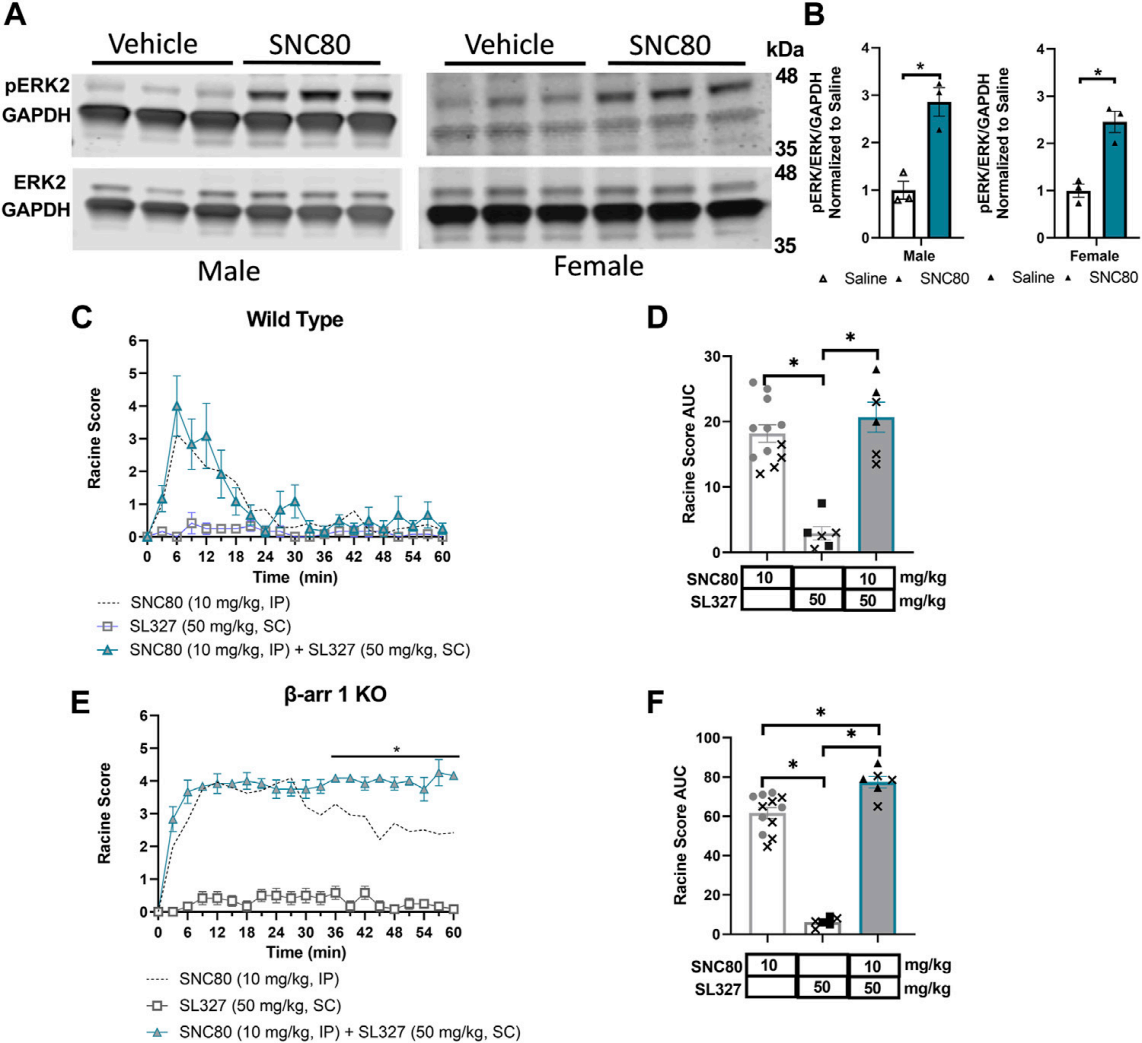
ERK activation by SNC80 does not cause seizures. **(A)** Representative western blot of SNC80 (10 mg/kg, i.p.)-induced activation of ERK in hippocampal tissue from male and female β-arrestin 1 KO mice relative to vehicle (n = 3 per treatment and sex). **(B)** Quantification of phosphorylated ERK (pERK) over total ERK (ERK) all normalized to GAPDH as loading control. Seizure activity over time **(C,E)** and as area under the curve (AUC) **(D,F)** in wild-type **(C,D)** or β-arrestin 1 KO **(E,F)** mice exposed to SNC80 (10 mg/kg, i.p.), SL327 (50 mg/kg i.p.) or SNC80 + SL327. Equal number of males and females tested (n = 6 total). *p < 0.05. Dotted line depicts 10 mg/kg SNC80 in wild-type **(C)** or β-arrestin 1 KO mice **(E)** for reference. In **(D,F)** male mice are depicted with a cross, whereas female mice are depicted as solid symbols.

Based on the strong correlation between β-arrestin 2 recruitment efficacy and seizure activity in mice only expressing β-arrestin 2 (i.e., β-arrestin 1 knockout mice), we next aimed to investigate potential mechanisms of action for δOR-agonist induced seizures as it relates to β-arrestin 2 signaling. Several kinases are known to scaffold with β-arrestin 2 including extracellular signal-related kinase (ERK). ERK signaling is involved with various cellular processes and has been shown to be stimulated downstream of δOR activation (Eisinger et al., 2002; Hong et al., 2009; Xu et al., 2010; Ko et al., 2021). Thus, we hypothesized that δOR-agonist induced activation of ERK in the hippocampus contributes to its seizure activity. We particularly focused on the hippocampus, for a multitude of reasons. First, SNC80-induced seizures have been linked to GABAergic neurons expressed in the forebrain (Chung et al., 2015), including the hippocampus. Second, the δOR is known to be expressed in the hippocampus and to mediate synaptic plasticity of hippocampal neurons (Valentino et al., 1982; Piskorowski and Chevaleyre, 2013; Erbs et al., 2014). Third, seizures caused by intrahippocampal injection of the δOR agonist Ala-deltorphin are mediated by the δOR (De Sarro et al., 1992), strongly implicating the hippocampus in δOR-agonist induced seizures. Additionally, earlier work done in our lab demonstrated that ERK is activated by SNC80 in the dorsal hippocampus and is attenuated in β-arrestin 2 knockout mice and amplified in β-arrestin 1 knockout mice (Ko et al., 2021). Western blot studies were performed to analyze phosphorylated ERK and total ERK in hippocampal tissue from β-arrestin 1 knockout male and female mice exposed to either vehicle (saline) or SNC80 (Figure 4A). Quantification of the data (Figure 4B) revealed that both male and female SNC80 exposed samples displayed significantly higher ratios of phosphorylated ERK compared to only vehicle exposed samples (2-way ANOVA, overall treatment effect *p* < 0.0001, F (1, 8) = 108.2), without a sex difference (2-way ANOVA, overall sex effect *p* = 0.09, F (1, 8) = 3.85). We note the caveat that the male and female samples were not run on the same blot and thus no firm conclusion can be drawn for sex differences. To test our hypothesis that δOR-agonist induced activation of ERK in the hippocampus contributes to its seizure activity, we utilized the MEK inhibitor, SL327, which indirectly but effectively inhibits ERK (Groblewski et al., 2011; Ko et al., 2021) in our mouse model. Following the same dosage and method of injection as used in our lab previously, male and female wild-type mice were exposed to 50 mg/kg SL327 30 min prior to a 10 mg/kg of SNC80 and subsequently recorded for 60 min. Another set of mice were tested with only SL327 as a negative control and recorded for 60 min starting 30 min after SL327 administration. We found that SL327 by itself does not induce seizures, as mice exposed to only SL327 maintained Racine scores close to zero (Figure 4C). Against our hypothesis, we found that SL327 did not decrease SNC80-induced seizures in wild-type mice (repeated measure 2-way ANOVA, overall treatment effect *p* < 0.0001, F (2, 21) = 29.05, with Tukey’s multiple comparison displaying no significant effect between ‘SL327 + SNC80’ to SNC80 at any time points) (Figure 4C). When analyzing area under the curve results, no statistically significant difference is seen between wild-type mice exposed to SNC80 in comparison to “SL327 + SNC80” (1-way ANOVA, *p* < 0.0001, F (2, 21) = 29.05, with Tukey’s multiple comparison “SL327 + SNC80” versus SNC80 *p* = 0.52) (Figure 4D). Though SL327 did not impact SNC80-induced seizures in wild-type mice, seeing as how β-arrestin 1 knockout mice are more sensitive to SNC80, we sought to analyze if SL327 would modify SNC80 seizure activity in β-arrestin 1 knockout mice. Following the same protocol as described earlier, we found that there is a significant increase in Racine scores reached between SL327 + SNC80-treated mice relative to SNC80-treated β-arrestin 1 knockout mice especially during the last 30 min (repeated measure 2-way ANOVA, overall treatment effect *p* < 0.0001, F (2, 21) = 143.1, with Tukey’s multiple comparison displaying a significant effect between “SL327 + SNC80” to SNC80 starting at the 36 min time point) (Figure 4E). Analysis over the entire 60 min, found a small but significant difference between “SL327 + SNC80” and SNC80 only group (1-way ANOVA, *p* < 0.0001, F (2, 21) = 143.1, with Tukey’s multiple comparison “SL327 + SNC80” versus SNC80 *p* < 0.01) (Figure 4F).

### 3.7 Honokiol dose-dependently inhibits SNC80-induced seizure activity in β-arrestin 1 knockout mice

**FIGURE 5:**
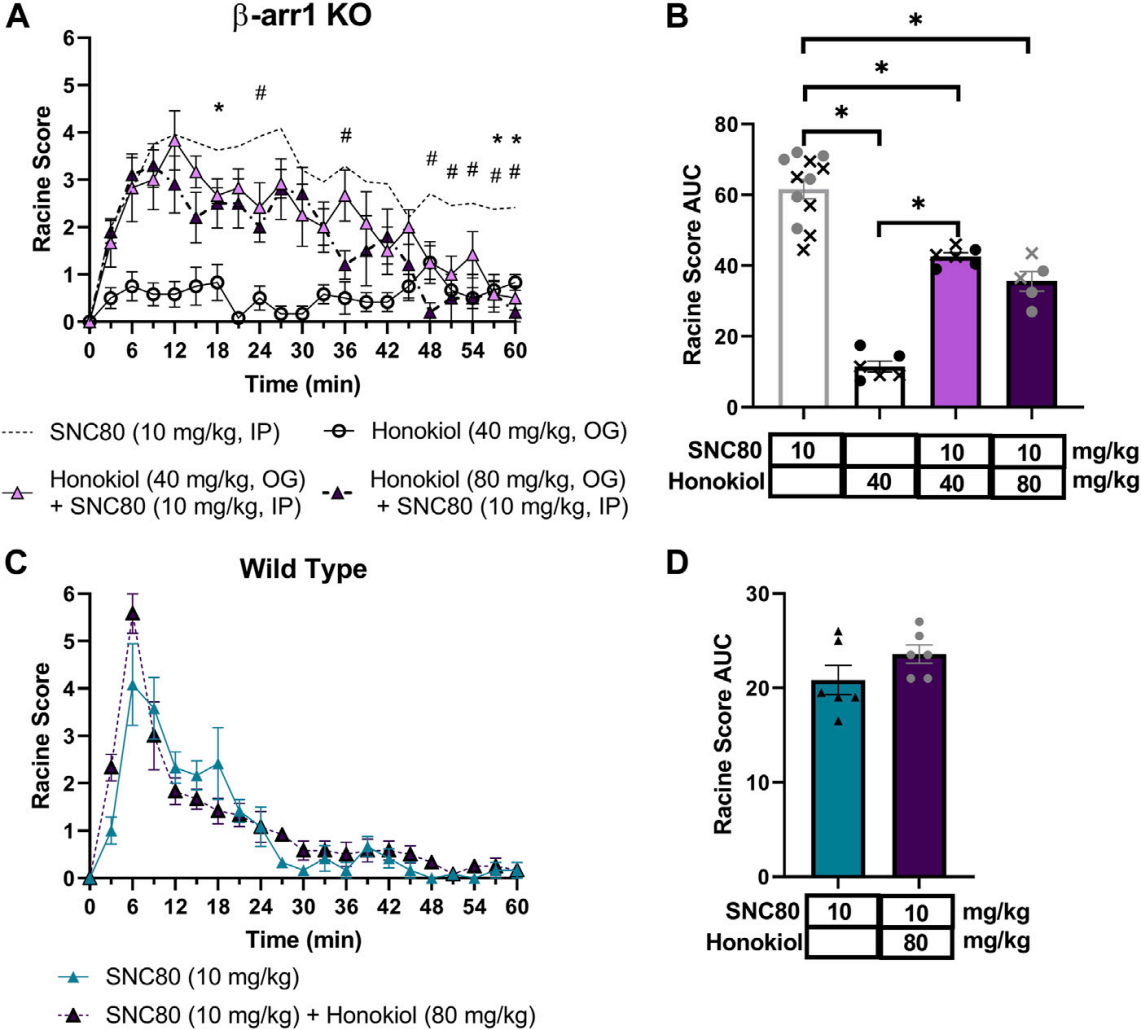
Honokiol dose-dependently reduces SNC80-induced seizures in β-arrestin 1 KO mice, but not in wild-type mice, and its seizure activity is attenuated by honokiol. Seizure activity over time **(A)** and as area under the curve (AUC) **(B)** in β-arrestin 1 KO or wild-type **(C,D)** mice exposed to SNC80 (10 mg/kg, i.p.), honokiol (40 mg/kg p.o.) or SNC80 + 40 or 80 mg/kg honokiol. [n = 5 (3 female and 2 male) for β-arrestin 1 KO mice and n = 6 female per treatment for wild-type mice]. *p < 0.05. Dotted line depicts 10 mg/kg SNC80 in β-arrestin 1 KO **(A)** or wild-type mice **(C)** for reference. In **(B)** male mice are depicted with a cross, whereas female mice are depicted as solid symbols.

We next hypothesized that δOR-agonist activation of the “mTOR pathway”, through a mechanism involving β-arrestin 2 scaffolding with AKT is responsible for their seizure activity. To determine this, we utilized the pI3K/AKT/mTOR pathway inhibitor honokiol, which has previously been used in mice and, importantly, can cross the blood brain barrier (Bai et al., 2003; Lin et al., 2012; Pillai et al., 2015; Fan et al., 2018; Park et al., 2020). We exposed β-arrestin 1 knockout mice to 40 or 80 mg/kg Honokiol 20 min prior to 10 mg/kg of SNC80. A separate group of mice received 40 mg/kg Honokiol followed by vehicle as a negative control. We observed that honokiol significantly shortened the duration of SNC80-induced seizure activity (repeated measure 2-way ANOVA, overall treatment effect *p* < 0.0001, F (3, 25) = 68.8 and treatment x time effect *p* < 0.0001, F (60, 500) = 2.81, with Tukey’s multiple comparison finding a significant difference between SNC80 and 40 mg/kg Honokiol + SNC80 at 18, 57 and 60 min, and between SNC80 and 80 mg/kg Honokiol + SNC80 to SNC80 at 24, 36 and 48–60 min) (Figure 5A). While there was no significant difference between the 40 and 80 mg/kg honokiol co-treatments (*p* = 0.23), there the larger number of occurrences for significant difference with 10 mg/kg SNC80 does suggest that 80 mg/kg was more potent at inhibiting SNC80-induced seizures Figure 5B). In wild-type mice, we did not find a clear significant effect for 80 mg/kg honokiol on SNC80-induced seizures (repeated measure 2-way ANOVA, overall treatment effect *p* = 0.16, F (1, 10) = 2.27 and a small treatment x time effect *p* < 0.05, F (20, 200) = 1.67, Figure 5C). The lack of an effect was also apparent when plotting the area under the curve (student t-test, *p* = 0.16, Figure 5D).

### 3.8 Seizures induced by 32 mg/kg SNC80 in wild-type mice is not affected by honokiol and is equally strong in the absence of β-arrestin 2 knockout

**FIGURE 6:**
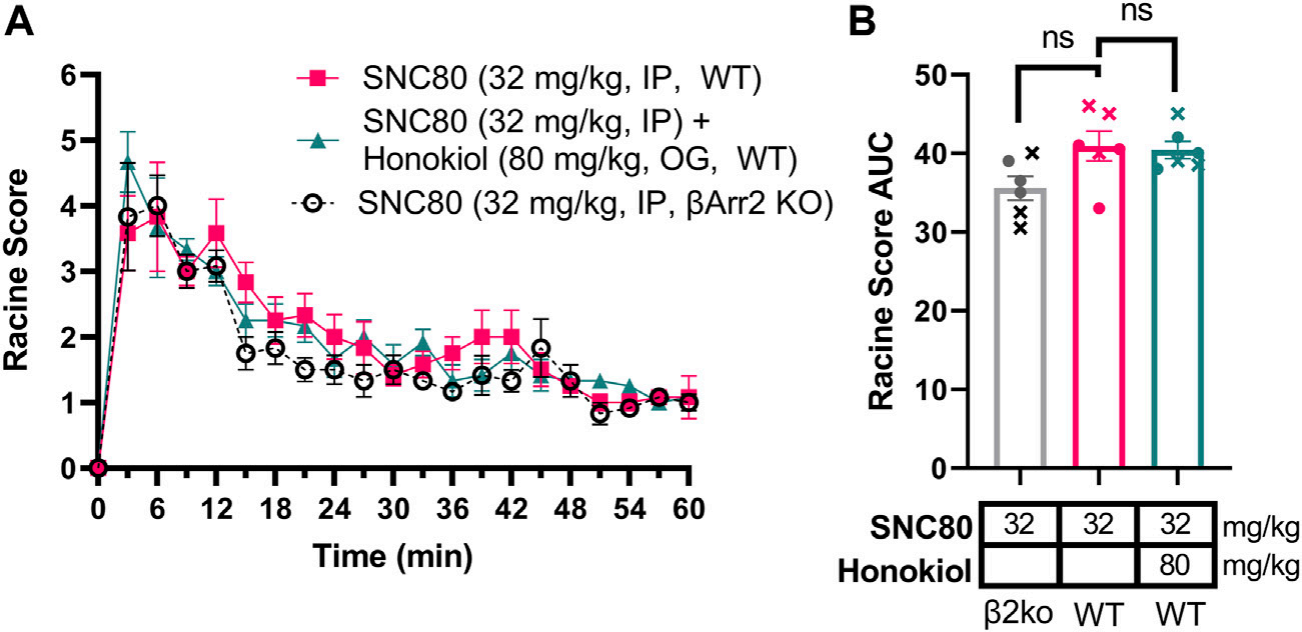
Stronger seizures induced by 32 mg/kg SNC80 in wild-type mice are not reduced by honokiol and similar in β-arrestin 2 knockout mice. Seizure activity over time **(A)** and as area under the curve (AUC) **(B)** in β-arrestin 2 KO or wild-type mice exposed to SNC80 (32 mg/kg, i.p.), or 32 mg/kg SNC80 and 80 mg/kg honokiol. [n = 6 (3 female and 3 male) in wild-type mice]. In **(B)** male mice are depicted with a cross, whereas female mice are depicted as circles.

Based on the discrepancy between the strong correlation of β-arrestin 2 recruitment and seizure intensity (Figure 3D) but the minimal difference in seizure intensity between wild-type and β-arrestin 2 knockout mice (Figure 2F), We hypothesized that β-arrestin 2 may only contribute to DOR agonist signaling and seizure activity at higher concentrations. To test this hypothesis, we exposed wild-type and β-arrestin 2 knockout mice to a 32 mg/kg dose of SNC80. This dose was chosen based on the previous studies showing that 32 mg/kg SNC80 produces similar seizure intensity in wild-type mice as does a 10 mg/kg SNC80 dose in β-arrestin 1 knockout mice (Vicente-Sanchez et al., 2018) (compare the E_max_ for 10 mg/kg SNC80 in Figure 2B with the E_max_ for 32 mg/kg SNC80 in Figure 6A). This dose of SNC80 indeed induced stronger ‘clonic’ seizure intensity (Racine score ≥4). However, we found no significant difference in 32 mg/kg SNC80 induced seizure activity between wild-type and β-arrestin 2 knockout mice [repeated measure 2-way ANOVA, treatment effect, F (2,15) = 3.73, *p* = 0.048 - post-hoc analysis did not find significant differences at any time-point; time × agonist interaction effect F (40, 300) = 0,072, *p* = 0.90] (Figures 6A,B). Given that honokiol was effective in reducing SNC80 seizure activity in β-arrestin 1 knockout mice, but not in wild-type mice, we similarly hypothesized that PI3K/AKT signaling may only be a component of δOR signaling at high agonist concentrations and significant seizure activity. However, the stronger seizure induced by 32 mg/kg SNC80 in wild-type mice was not impacted by treatment with 80 mg/kg honokiol (Figures 6A,B), which agreed with findings by Dripps and co-workers (Dripps et al., 2018).

### 3.9 PQR530 does not inhibit SNC80-induced seizures in β-arrestin 1 knockout mice

**FIGURE 7:**
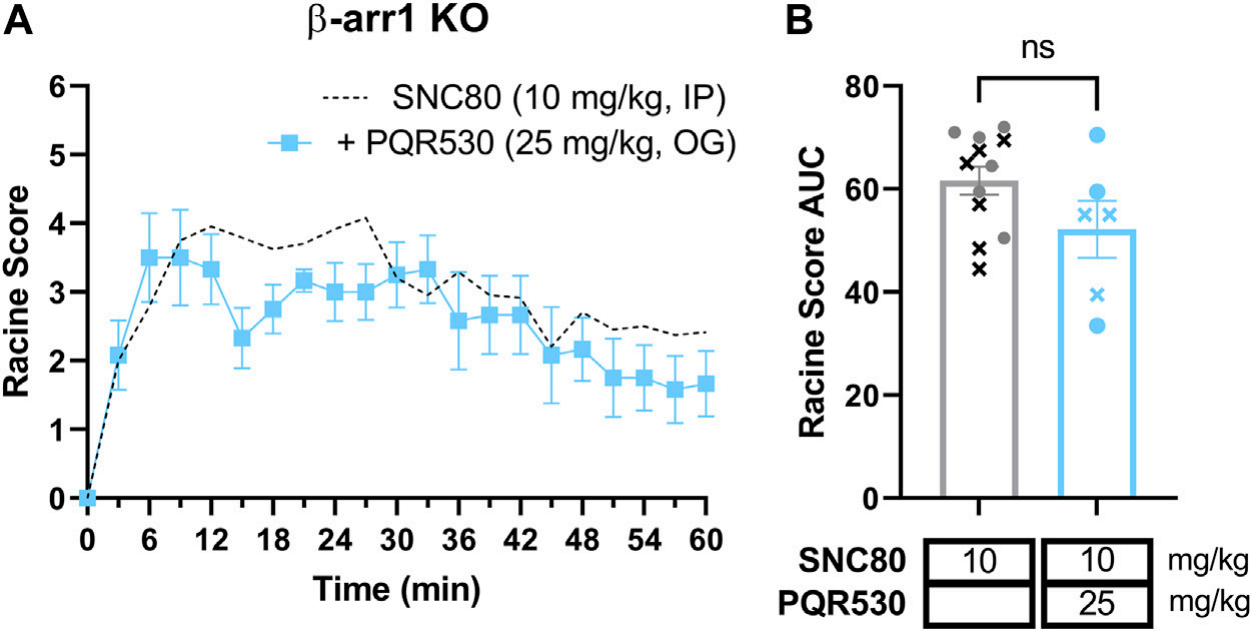
The PI3K and mTOR inhibitor PQR530 does not inhibit SNC80-induced seizures in β-arrestin 1 knockout mice. Seizure activity over time **(A)** and as area under the curve (AUC) **(B)** in β-arrestin 1 KO exposed to SNC80 (10 mg/kg, i.p.) and 25 mg/kg PQR530 [n = 6 (3 female and 3 male)]. Dotted line depicts 10 mg/kg SNC80 in β-arrestin 1 KO for reference. In **(B)** male mice are depicted with a cross, whereas female mice are depicted as circles.

In the absence of reproducible antibodies in the PI3K pathway, we opted to confirm the role of the PI3K/AKT/mTOR pathway in β-arrestin 1 knockout mice using a different inhibitor. We chose to administer β-arrestin 1 knockout mice with 25 mg/kg PQR530, based on reports that this inhibitor of PI3K/mTOR is brain penetrable (Brandt et al., 2018; Rageot et al., 2019) and was effective in mice at this dose (Rageot et al., 2019; Theilmann et al., 2020). However, in our hands PQR530 given p. o. 30 min before a 10 mg/kg SNC80 dose did not alter seizure responses [repeated measure 2-way ANOVA, agonist effect, F (1,16) = 3.00, *p* = 0.10 and time × agonist interaction effect F (20, 320) = 1.20, *p* = 0.26] (Figures 7A,B).

## 4 Discussion

We had hypothesized that β-arrestins were involved in the induction of seizures by certain δOR agonists as those δOR agonists producing the strongest seizures are known to efficaciously recruit β-arrestin (Comer et al., 1993; Broom et al., 2002a; Jutkiewicz et al., 2004; Jutkiewicz et al., 2005a; Jutkiewicz et al., 2005b) and because β-arrestin 1 knockout mice display a potentiated seizure response following SNC80 administration (Vicente-Sanchez et al., 2018). The importance for β-arrestin, or inversly the relatively weak role for G-protein signaling in SNC80 induced seizures could also be deduced from a study using regulator of G-protein signaling (RGS) 4 knockout mice. Unsurprisingly in these mice, as RGS promotes the inactivation of the G-protein, SNC80 exhibited enhanced antinociceptive/anti-allodynic and anti-depressive-like effects for SNC80. Yet, SNC80-induced seizure activity was unaltered in the RGS4 knockout mice, suggesting that SNC80-induced seizure activity was independent of G-protein signaling (Dripps et al., 2017). Furthermore, δOR agonists such as KNT-127, 7OH-speciogynine, TRV250, and PN6047 all of which under-recruit β-arrestin 2 relative to Leu-enkephalin, and may be considered G-protein-biased, do not cause seizures, further suggesting that G-protein signaling has a negligible contribution to the seizure mechanism (Saitoh et al., 2011; Conibear et al., 2020; Fossler et al., 2020; Gutridge et al., 2021). In contrast, δOR agonists, like SNC162 and BW373U86, that strongly recruit β-arrestin (Fenalti et al., 2014; Chiang et al., 2016) do induce seizures (Comer et al., 1993; Broom et al., 2002a; Jutkiewicz et al., 2004; Jutkiewicz et al., 2005a; Jutkiewicz et al., 2005b). In this study cellular pharmacology findings did correlate β-arrestin 2 recruitment efficacy of δOR agonists with seizure intensity, yet we did find that SNC80 induced seizures in wild-type mice at 10 and 32 mg/kg doses were identical in β-arrestin 2 knockout mice, in agreement with previous findings (Dripps et al., 2018). It is possible that congenital global knockout of β-arrestin 2 is not a good system to assess mechanisms that occur in wild-type mice. This appears to be the case for the β-arrestin 1 knockout mice, as will be discussed in more detail below and exemplified by our finding that only in β-arrestin 1 knockout mice, but not wild-type mice, SNC80-induced seizures were modulated by inhibition of ERK or PI3K/AKT signaling.

We initially hypothesized that β-arrestin 1 was protective against δOR-induced seizures given that β-arrestin 1 knockout mice exhibit increased seizure sensitivity compared to wild type mice in response to SNC80 (Vicente-Sanchez et al., 2018). However, if β-arrestin 1 signaling was attenuating seizure activity in wild-type mice, and G-protein signaling has no meaningful contribution, then theoretically β-arrestin 2 knockout mice should not exhibit seizures in response to δOR agonists, as only G-protein and β-arrestin 1 signaling are available. Yet this is not the case, because, while in our study βarrestin 1 knockout mice do show enhanced sensitivity to seizures, wild-type and β-arrestin 2 knockout mice show comparable seizure behavior in response to various δOR agonists. Therefore, an alternative interpretation of our data is that β-arrestin 2 promotes seizures because β-arrestin 1 knockout mice, which only express β-arrestin 2, show a higher propensity towards seizures and because Racine score was found to strongly correlate specifically with the β-arrestin 2 recruitment efficacy of each tested δOR agonist (Figure 3D). Still, under this hypothesis, one would expect β-arrestin 2 knockout mice to be devoid of δOR seizures. A possible explanation for the finding that wild-type and β-arrestin 2 knockout mice are indistinguishable is that while SNC80 can strongly promote δOR to interact with both β-arrestin isoforms, SNC80 preferentially promotes δOR to interact with β-arrestin 1 (Pradhan et al., 2016). Thus, in wild-type and β-arrestin 2 knockout mice, SNC80 will predominantly promote the recruitment of β-arrestin 1. Genetic knockout of both β-arrestin isoforms is embryonically lethal and this limits the ability to assess if mice lacking β-arrestin 1 and β-arrestin 2 would not produce seizures in response to SNC80. A potentially simpler explanation of our results is that knockout of β-arrestin 1 leads to enhanced receptor externalization in response to SNC80 (Pradhan et al., 2016), then the enhanced seizure activity in β-arrestin 1 knockout mice could be due to enhanced δOR expression. This hypothesis would also involve an initial condition of low receptor reserve in which ‘G-protein-biased’ δOR agonists are partial agonists that limit their ability to induce seizures. There has been significant debate regarding µOR biased agonism, that G-protein-biased µOR agonists may lack certain side effects not because of their preference for G-protein signaling over β-arrestin recruitment, but instead due to low intrinsic efficacy (Gillis et al., 2020). However, this hypothesis is less likely per a study that showed that BU48, a weak δOR partial agonist, induced seizures (Dripps et al., 2020) and per the aforementioned RGS4 knockout study, in which increasing SNC80 potency/efficacy did not increase seizures (Dripps et al., 2017).

Thus, we explored the hypothesis that β-arrestin 2 contributes to severe seizure activity and to investigate if kinases known to scaffold with β-arrestin 2 could be linked to δOR agonist-induced seizure. We decided to use both the wild-type and β-arrestin 1 knockout mice, the latter as a model system with reduced complexity of the cellular environment by ensuring cells only express β-arrestin 2. For this part of the study, we narrowed our approach by only testing SNC80 and by selecting the hippocampus to investigate kinase activity. The rationale, was that microinjection of various δOR agonists, including SNC80, deltorphin, and DSTLE, into the dorsal and ventral hippocampal areas produced convulsive behavior and epileptic-like EEG recordings (Haffmans and Dzoljic, 1983; De Sarro et al., 1992; Sakamoto et al., 2021) suggesting that activation of δOR in the hippocampus was sufficient to produce seizures. Still, this is a significant limitation of our study, and we do not rule out that other regions may also be involved. The cortex particularly may be of interest as some studies of δOR agonist-induced seizures have found changes in electrocorticography recordings (Haffmans and Dzoljic, 1987; De Sarro et al., 1992), which are EEG studies specifically recording activity of the cortex, and knockout of δOR in GABA-ergic forebrain (which includes the cortex) neurons prevented SNC80-induced seizures (Chung et al., 2015). Future work is required to more broadly study signaling induced by seizurogenic δOR agonists across brain areas.

We were particularly interested in studying ERK and PI3K/AKT signaling, as these are known pathways to be modulated by δOR selective agonists in the central nervous system (Ko et al., 2021; Kawaminami et al., 2022). We first investigated pERK, based on prior evidence indicating that SNC80 increased pERK in wild-type mice, and importantly that pERK activation was enhanced in β-arrestin 1 knockout mice (Ko et al., 2021). Multiple studies have implicated ERK signaling in the development of epileptic seizures, potentially via activation of NMDA receptors (Nateri et al., 2007; Pernice et al., 2016; Nguyen et al., 2022). Yet, we found that indirect inhibition of ERK did not attenuate SNC80-induced seizures in wild-type mice and actually prolonged seizures in β-arrestin 1 knockout mice. It is possible that ERK is modulating mTOR signaling (Roux et al., 2007; Gautam et al., 2021). In that respect, AKT can also be activated through β-arrestin 2 and induce mTOR activation (Kendall et al., 2014) is part of the larger P13K/AKT/mTOR pathway, a vastly influential, multifaceted signaling pathway crucial for cell growth and survival (Porta et al., 2014), although by our knowledge this pathway has not been established at the δOR. This ‘mTOR’ pathway, is appreciated for its role in epilepsy, and the mTOR protein complex specifically has been the target of anti-epilepsy or anti-seizure drugs, with many mTOR inhibitors effectively preventing seizures (Wong, 2013; Griffith and Wong, 2018). β-arrestin proteins may even directly scaffold with S6K or p70 ribosomal S6 kinase, a downstream effector of mTOR (Trefier et al., 2018). There has been significant controversy regarding antibodies directed at the mTOR pathway proteins (Schneider, 2020), To circumvent this, we opted for a pharmacological approach of studying this pathways’ possible involvement in δOR-mediated seizures using a broad brain-penetrable inhibitor of the pathway. We find that honokiol attenuates SNC80-induced seizure activity in β-arrestin 1 knockout mice, reducing seizure effect by nearly 50% at the highest dose tested (Racine score area under the curve 61.6 versus 35.6). However, honokiol did not significantly attenuate SNC80 seizures in wild-type mice. In β-arrestin 1 knockout mice honokiol primarily reduced the duration and not the intensity of SNC80-induced seizures. Seizure activity in β-arrestin 1 knockout mice treated with 10 mg/kg SNC80 and 80 mg/kg honokiol was similar to wild-type and β-arrestin 2 knockout mice treated with 32 mg/kg SNC80.

Under most conditions SNC80 induces seizures, however in rats 30 mg/kg SNC80 reportedly has anti-convulsant activity in reducing pilocarpine-induced seizures (Bausch et al., 2005), highlighting the intricacies involved in δOR function and seizure activity. Still, the pro-convulsant effects of SNC80 are absent in δOR knockout mice (Broom et al., 2002b; Chung et al., 2015), suggesting that effect is on-target. It is however possible that the seizure activity is mediated by a distinct target that is modulated by the δOR via cross talk. For example, SNC80 can inhibit calcium and potassium current and promote sodium currents in cultured hippocampal rat neurons (Moravcikova et al., 2021). SNC80 also can increase extracellular glutamate levels by inhibition of striatal GABA transmission, resulting in downstream NMDA activation (Bosse et al., 2014). It is noteworthy that (-)TAN-67 can also induce NMDA activation (Fusa et al., 2005), yet does not induce seizures.

Lastly, it is also necessary to call attention to the potential sex differences seen with these seizures. Our 3-h study found that male β-arrestin 1 knockout mice had stronger seizures compared to female mice in response to SNC80; but no difference was seen within the first hour, which was why our later studies only analyzed the first hour post drug exposure. Prior studies have indicated sex differences for δOR signaling in hippocampal areas (Harte-Hargrove et al., 2015; Mazid et al., 2016). Previous studies have also shown that female mice are more likely to show enhanced AKT phosphorylation, specifically in the hippocampus, in response to estrogen (Sheppard et al., 2021). If this is the case, then it is possible that inhibiting AKT with honokiol in male β-arrestin 1 knockout mice may have less of an effect on SNC80-induced seizures. We did notice that the strongest decrease in Racine score occurred in two female mice (Figure 5B), however, we did not explore this in detail by increasing our group size.

## 5 Conclusion

**FIGURE 8:**
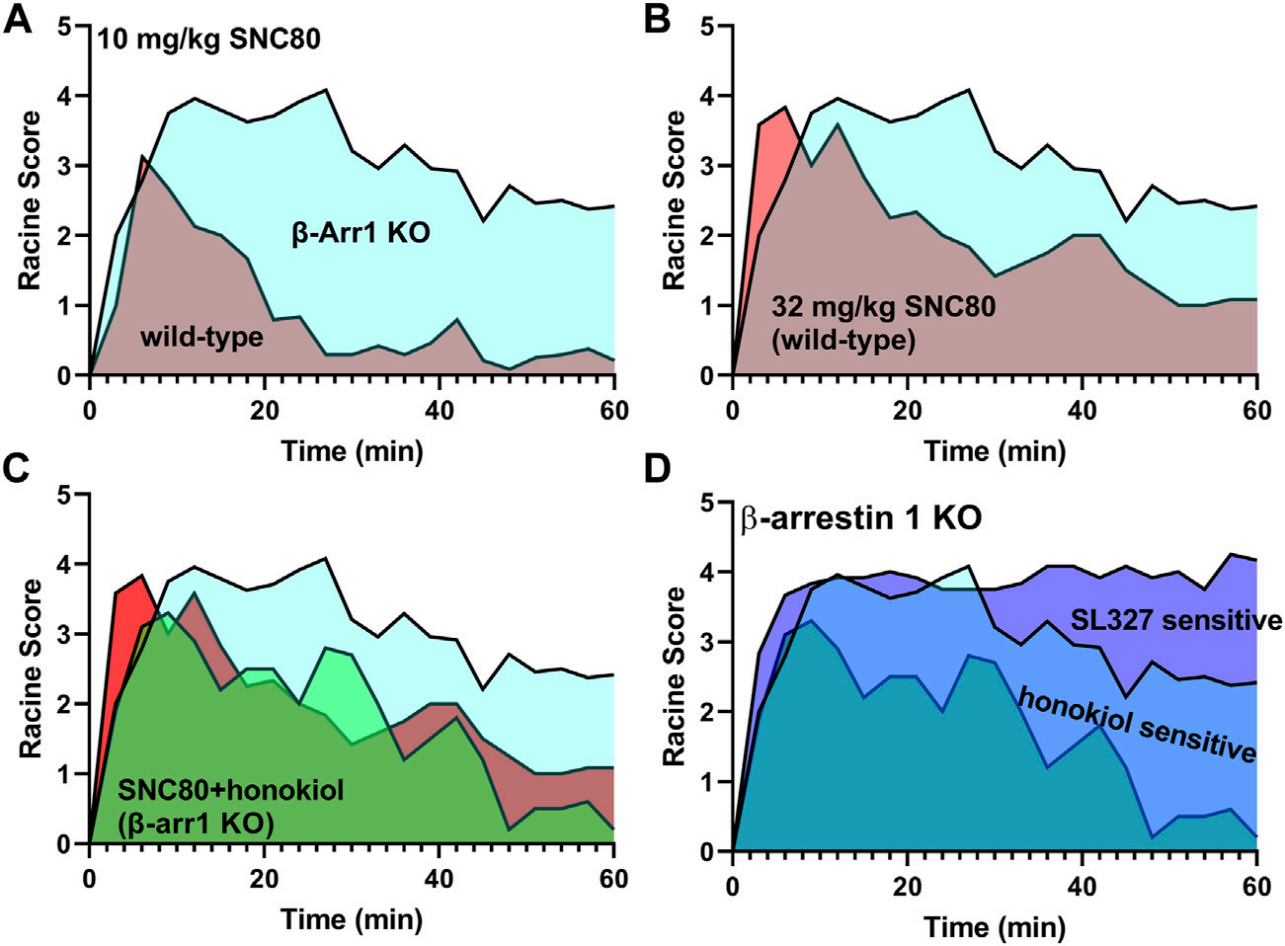
Enhanced δOR-agonist induced seizures in β-arrestin 1 knockout mice are modulated by ERK and AKT inhibition, while those kinases are not part of δOR-induced seizures in wild-type mice. 10 mg/kg SNC80 produces more severe seizures in β-arrestin 1 knockout mice compared to wild-type mice **(A)**. 32 mg/kg SNC80 in wild-type mice produces clonic seizures (Racine score 4), similar to 10 mg/kg SNC80 in β-arrestin 1 knockout mice, but the intensity tapers off more rapidly in wild-type mice **(B)**. Inhibition of PI3K/AKT reduces seizure effects for 10 mg/kg SNC80 in β-arrestin 1 knockout mice to levels comparable to 32 mg/kg SNC80 in wild-type mice **(C)**. The component of enhanced seizure effect observed for 10 mg/kg SNC80 in β-arrestin 1 knockout mice is sensitive to modulation by ERK and PI3K/AKT inhibition **(D)**.

In conclusion, β-arrestin 1 knockout mice show a sensitized response in seizure effects (Figure 8A). A higher dose of SNC80 (32 mg/kg) in wild-type can match the intensity but not duration of seizure effects obsevered in β-arrestin 1 knockout mice (Figure 8B). Honokiol reduced SNC80 seizures in β-arrestin 1 knockout mice, but could not reduce it below the seizure activity levels produced by 32 mg/kg SNC80 in wild-type mice (Figure 8C). Therefore, we propose that β-arrestin 1 knockout mice exhibit compensatory changes that are susceptible to modulation of ERK and PI3K/AKT signaling pathways (Figure 8D), but this mechanism is not physiologically relevant as neither modulation of ERK or PI3K/AKT alters SNC80-induced seizure activity. Altogether, our work suggests that it is prudent to limit β-arrestin recruitment efficacy for a clinical trial δOR agonist candidate to reduce the risk of seizure activity but that β-arrestin 1 knockout mice are not a physiologically relevant model system to investigate the mechanism of action of δOR-agonist induced seizures.

## Data Availability

The raw data supporting the conclusions of this article will be made available by the authors, without undue reservation.
